# A phase Ib study of TQB2450 plus anlotinib in patients with advanced triple-negative breast cancer

**DOI:** 10.1016/j.isci.2023.106876

**Published:** 2023-05-13

**Authors:** Jiayu Wang, Tao Sun, Quchang Ouyang, Yiqun Han, Binghe Xu

**Affiliations:** 1 Department of Medical Oncology, National Cancer Center/National Clinical Research Center for Cancer/Cancer Hospital, Chinese Academy of Medical Sciences and Peking Union Medical College, Beijing, China; 2 Liaoning Cancer Hospital & Institute, Shenyang, China; 3 Department of Breast Cancer Medical Oncology, Hunan Cancer Hospital, Changsha, China

**Keywords:** Clinical medicine, Health sciences, Oncology

## Abstract

This study explored the safety and preliminary efficacy of the anti-PD-L1 antibody TQB2450 combined with the multi-kinase inhibitor anlotinib in advanced triple-negative breast cancer (TNBC). Patients with advanced TNBC who received at least one line of systemic therapy with anthracyclines and/or taxanes were enrolled in the dose-escalation and dose-expansion cohorts. Between May 29, 2019 and September 28, 2020, 34 patients were enrolled (three in the dose-escalation cohort and 31 in the dose-expansion cohort). The ORR was 26.5% (95% CI, 12.9–44.4) and the DCR was 73.5% (95% CI, 55.6–87.1). The median PFS was 5.6 (95% CI, 2.9–7.5) months, and the median OS was not reached. Seventeen (50.0%) patients had grade ≥3 treatment-related adverse events, with the most common being QT interval prolongation (17.6%) and hypertension (14.7%). No treatment-related deaths occurred. TQB2450 combined with anlotinib as a chemotherapy-free treatment shows promising efficacy with a manageable safety profile for patients with previously treated advanced TNBC.

## Highlights

• TQB2450 + anlotinib shows promising efficacy in previously treated advanced TNBC
• TQB2450 + anlotinib is the first anti-PD-L1 + antiangiogenic drug regimen for aTNBC
• TQB2450 + anlotinib shows a manageable safety profile in such patients

## Graphical abstract

**Figure:**
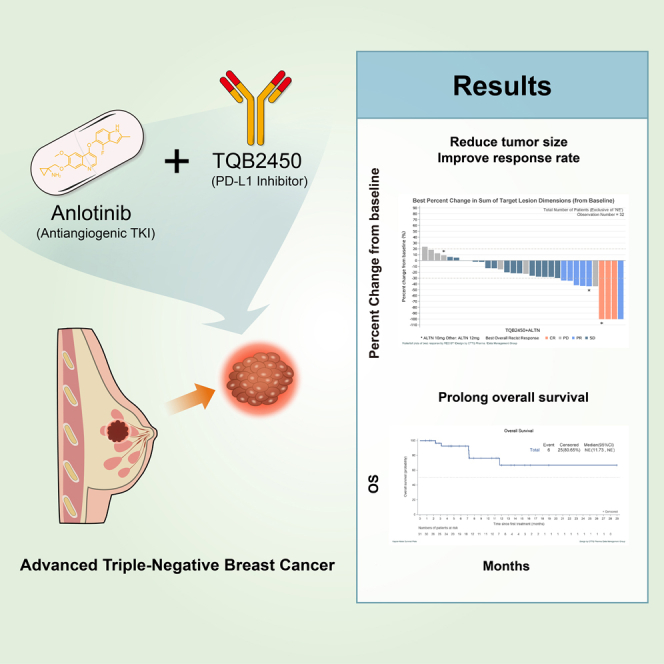

## Introduction

Triple-negative breast cancer (TNBC) is a type of breast cancer characterized by the absence of estrogen receptor (ER), progesterone receptor (PR) and human epidermal growth factor receptor 2 (HER2) expression.^1^ TNBC accounts for approximately 15% of all breast cancers,^2^^,^^3^ and its lack of druggable targets limits the clinical benefits of endocrine or targeted therapy for TNBC patients. Consequently, TNBC tends to be more aggressive and have a higher risk of metastasis, leading to worse prognosis compared to other breast cancer subtypes.^4^^,^^5^ Indeed, first-line chemotherapy and immunotherapy can only provide clinical benefits in a small number of patients with advanced TNBC. In September 2020, the Food and Drug Administration (FDA) warned that atezolizumab plus paclitaxel was not applicable for the first-line treatment of advanced TNBC.^6^ Despite advances in first-line treatment options for advanced TNBC,^7^^,^^8^^,^^9^^,^^10^ chemotherapy with modest efficacy remains the mainstay of treatment in the second- or further-line setting.^11^^,^^12^^,^^13^ A previous phase III trial demonstrated that immunotherapy with pembrolizumab alone could not significantly improve overall survival (OS) in patients with previously treated metastatic TNBC compared to chemotherapy.^14^ New agents or combination regimens are urgently needed for advanced TNBC, particularly in the second- or further-line setting.

Immunotherapy is currently a promising research area of advanced cancer treatment. Immune checkpoint inhibitor (ICI) plus chemotherapy showed significant improvement in progression-free survival (PFS) among patients with advanced TNBC compared to chemotherapy alone,^15^ which was approved by the FDA for the first-line treatment of advanced TNBC and proved the promising efficacy of ICIs in patients with TNBC. Furthermore, tumor angiogenesis is known to play a critical role in tumor growth and invasion.^16^^,^^17^ The chemotherapy-free regimen of an ICI plus an antiangiogenic drug has shown promising efficacy in endometrial and ovarian tumors.^18^^,^^19^ A phase II trial investigated the use of camrelizumab plus apatinib in patients with advanced TNBC, including 75% (30/40) who had received no more than one line of prior therapy. Although the safety profile was manageable, the survival benefit was still unsatisfactory.^20^ In addition, there are currently no studies on a programmed cell death protein ligand 1 (PD-L1) inhibitor plus an antiangiogenic drug for advanced TNBC.

Anlotinib is a novel small-molecule tyrosine kinase inhibitor (TKI) that mainly targets vascular endothelial growth factor receptor 1–3 (VEGFR 1–3), fibroblast growth factor receptor 1–4 (FGFR 1–4), platelet-derived growth factor receptor-alpha (PDGFR-α), platelet-derived growth factor receptor-beta (PDGFR-β), and the stem cell factor receptor c-KIT to inhibit tumor angiogenesis and growth.^21^^,^^22^^,^^23^ In a phase II single-arm clinical trial, anlotinib resulted in a PFS of 4.04 months with tolerable toxicity in patients with TNBC who had received multiple lines of therapy.^24^

TQB2450 is a humanized monoclonal antibody that targets PD-L1. Preliminary results from a phase I pharmacokinetic study (CTR20180272) suggested that patients with advanced diffuse large B-cell lymphoma could benefit from TQB2450 treatment. This preliminary activity of TQB2450 in the treatment of advanced malignant tumors warranted further investigations. For subsequent trials, the recommended dose of TQB2450 was 1200 mg once every 3 weeks. Mechanistically, we speculated that TQB2450 in combination with anlotinib could have a synergistic effect in treating advanced TNBC. Meanwhile, patients might better tolerate chemotherapy-free combination therapy than chemotherapy.

This phase Ib trial aimed to explore the safety and preliminary efficacy of PD-L1 inhibitor TQB2450 combined with anlotinib in patients with advanced TNBC.

## Results

### Baseline characteristics of the patients

Between May 29, 2019 and September 28, 2020, a total of 34 patients with advanced TNBC were enrolled in the study. Three patients in the dose-escalation phase who received 10 mg anlotinib plus 1200 mg TQB2450, as well as three patients with 12 mg anlotinib plus TQB2450, experienced no dose-limiting toxicities (DLTs) in the first cycle. Consequently, 28 patients with advanced TNBC received 12 mg of anlotinib plus TQB2450 in the expansion phase. Table 1 shows the baseline characteristics of the patients, with a median age of 50.0 (range, 32.0–70.0) years, and 76.5% of the patients having received only one line of prior systemic therapy. Twenty-eight (82.4%) patients had at least two metastatic sites, with 20 patients having visceral metastases, including the lung (35.3%) and the liver (23.5%) metastases. Six (17.6%) patients had positive PD-L1 expression. As of January 30, 2022, four patients had withdrawn from the study due to patient decision, 27 had disease progression, and three patients were still on treatment. The median follow-up was 8.79 (range, 5.78–13.40) months.

**Table 1:** Baseline characteristics

Characteristics	Total (N = 34)	TQB2450 1200 mg+anlotinib 10 mg (N = 3)	TQB2450 1200 mg+anlotinib 12 mg (N = 31)
Age (years), median (range)	50.0 (32.0, 70.0)	48.0 (48.0, 64.0)	50.0 (32.0, 70.0)
<65, n (%)	31 (91.2)	3 (100)	28 (90.3)
≥65, n (%)	3 (8.8)	0	3 (9.7)
ECOG performance status, n (%)			
0	30 (88.2)	3 (100)	27 (87.1)
1	4 (11.8)	0	4 (12.9)
Metastatic site, n (%)			
Non-visceral	30 (88.2)	3 (100)	27 (87.1)
Visceral	20 (58.8)	0	20 (64.5)
Number of metastatic sites, n (%)			
1	6 (17.7)	2 (66.7)	4 (12.9)
2	13 (38.2)	1 (33.3)	12 (38.7)
≥3	15 (44.12)	0	15 (48.4)
Chest wall metastasis, n (%)	10 (29.4)	2 (66.7)	8 (25.8)
Liver metastasis, n (%)	8 (23.5)	0	8 (25.8)
Bone metastasis, n (%)	10 (29.4)	0	10 (32.3)
Lung metastasis, n (%)	12 (35.3)	0	12 (38.7)
Lymph nodes metastasis, n (%)	24 (70.6)	2 (66.7)	22 (71.0)
Pleural effusion metastasis, n (%)	3 (8.8)	0	3 (9.7)
Pericardium metastasis, n (%)	0	0	0

Previous chemotherapy, n (%)			

Anthracyclines	32 (94.1)	3 (100)	29 (93.5)
Taxanes	31 (91.2)	3 (100)	28 (90.3)
Platinum	16 (47.1)	2 (66.7)	14 (45.2)
Previous neoadjuvant therapy, n (%)	9 (26.5)	1 (33.3)	8 (25.8)
Previous adjuvant therapy, n (%)	25 (73.5)	3 (100)	22 (71.0)
Number of previous therapy lines			
Median (range)	1 (0, 4)	1 (1, 1)	1 (0, 4)
0, n (%)	1 (2.9)	0	1 (3.2)
1, n (%)	26 (76.5)	3 (100)	23 (74.2)
2, n (%)	4 (11.8)	0	4 (12.9)
>2, n (%)	3 (8.8)	0	3 (9.7)
PD-L1 expression, n (%)			
Negative	13 (38.2)	1 (33.3)	12 (38.7)
Positive	6 (17.6)	1 (33.3)	5 (16.1)
Unknown	15 (44.1)	1 (33.3)	14 (45.2)

ECOG, Eastern Cooperative Oncology Group; PD-L1, programmed cell death-ligand 1. Demographics were summarized with descriptive statistics.

### Efficacy

**Figure 1:**
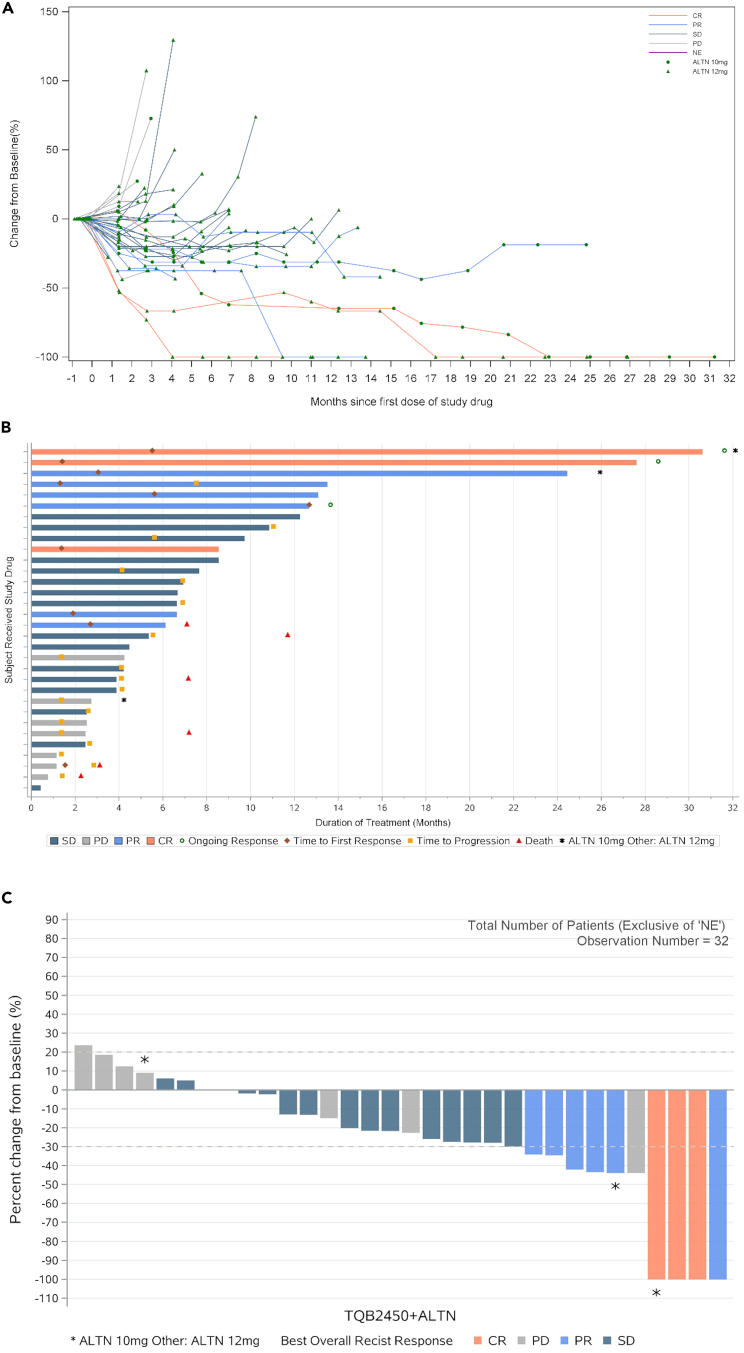
Tumor response in individual patients (A) Spider plot for the duration of response. (B) Swimming plot for the duration of response. (C) Waterfall plot for best change in tumor size from baseline. CR, complete response; PR, partial response; SD, stable disease; PD, progressive disease; NE, not evaluable.

As shown in Table 2, three (8.8%) out of the 34 patients achieved a complete response (CR), while six (17.6%) achieved a partial response (PR), resulting in an objective response rate (ORR) of 26.5% (95% confidence interval [CI], 12.9–44.4). The disease control rate (DCR) was 73.5% (95% CI, 55.6–87.1). The rates of progressive disease in the total population, patients who received 10 mg of anlotinib, and patients who received 12 mg of anlotinib were 20.6%, 33.3%, and 19.4%, respectively. Figure 1 shows the spider and swimming plots for the duration of response (DoR) and the waterfall plots for the best change in tumor size from baseline.

**Table 2:** Tumor response

Response	Total(N = 34)	TQB2450 1200 mg +anlotinib 10 mg (N = 3)	TQB2450 1200 mg +anlotinib 12 mg (N = 31)
**Best response, n (%)**			

CR	3 (8.8)	1 (33.3)	2 (6.5)
PR	6 (17.6)	1 (33.3)	5 (16.1)
SD	16 (47.1)	0	16 (51.6)
PD	7 (20.6)	1 (33.3)	6 (19.4)
NE	2 (5.9)	0	2 (6.5)
ORR, % (95% CI)	26.5 (12.9, 44.4)	66.7 (9.4, 99.2)	22.6 (9.6, 41.1)
DCR, % (95% CI)	73.5 (55.6, 87.1)	66.7 (9.4, 99.2)	74.2 (55.4, 88.1)

CR, complete response; PR, partial response; SD, stable disease; PD, progressive disease; NE, not evaluable; ORR, objective response rate; CI, confidence interval; DCR, disease control rate. ORR and DCR were summarized with descriptive statistics. The 95% confidence intervals (CIs) of ORR and DCR were calculated using the Clopper-Pearson method.

**Figure 2:**
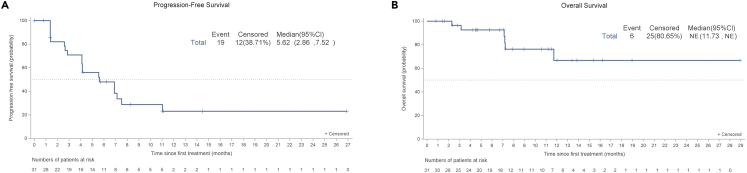
Kaplan-Meier curves of survival (A) Kaplan-Meier curve of progression-free survival (PFS). (B) Kaplan-Meier curve of overall survival (OS). PFS and OS were estimated with the Kaplan-Meier method.

The median PFS was 5.6 (95% CI, 2.9–7.5) months, and the median DoR was not reached (95% CI, 4.5-not reached). The PFS rate at 6 months in the total population was 49.9% (95% CI, 31.1–66.1). Patients with CR or PR had a longer median PFS than those with stable disease (not reached [95% CI, 7.1-not reached] vs. 5.6 [95% CI, 4.1–6.9] months, p = 0.001). The median OS was not reached (95% CI, 11.7-not reached), as depicted in Figure 2.

### Subgroup analysis of PFS

Among the 34 patients enrolled in the study, six were PD-L1 positive, and 13 were PD-L1 negative. The median PFS in PD-L1-positive patients (5.6 [95% CI, 1.38-not reached] months) was numerically lower than that in PD-L1-negative patients (11.0 [95% CI, 2.6-not reached] months) (Figure S1). The PFS rate at 6 months for PD-L1-positive and PD-L1-negative patients was 44.4% (95% CI, 6.6–78.5) and 68.6% (95% CI, 30.5–88.7), respectively.

### Safety

No DLTs were observed in the dose-escalation cohort. Among the 34 patients, 17 (50.0%) experienced grade ≥3 treatment-related adverse events (TRAEs) (Table 3). Grade ≥3 TRAEs included QT interval prolongation (n = 6 [17.6%]), hypertension (n = 5 [14.7%]), hand-foot syndrome (n = 3 [8.8%]), diarrhea (n = 3 [8.8%]), hypertriglyceridemia (n = 2 [5.9%]), hypercholesterolemia (n = 1 [2.9%]), decreased neutrophil count (n = 1 [2.9%]), oral mucositis (n = 1 [2.9%]), loss of appetite (n = 1 [2.9%]), weight loss (n = 1 [2.9%]), increased alanine aminotransferase (ALT) (n = 1 [2.9%]), increased aspartate aminotransferase (AST) (n = 1 [2.9%]), decreased white blood cell (n = 1 [2.9%]), abdominal pain (n = 1 [2.9%]), and increased blood bilirubin (n = 1 [2.9%]) (Table 4). No treatment-related deaths were reported.

**Table 3:** Summary of adverse events

Event, n (%)	Total (N = 34)	TQB2450 1200 mg +anlotinib 10 mg (N = 3)	TQB2450 1200 mg +anlotinib 12 mg (N = 31)
SAE	3 (8.8)	0	3 (9.7)
Any TRAE	34 (100)	3 (100)	31 (100)
Anlotinib-related	34 (100)	3 (100)	31 (100)
TQB2450-related	34 (100)	3 (100)	31 (100)
Grade ≥3 TRAE	17 (50.0)	2 (66.7)	15 (48.4)
Anlotinib-related	17 (50.0)	2 (66.7)	15 (48.4)
TQB2450-related	15 (44.1)	2 (66.7)	13 (41.9)

TEAE, treatment-emergent adverse event; SAE, serious adverse event; TRAE, treatment-related adverse event. AEs were summarized with descriptive statistics.

**Table 4:** Treatment-related adverse events (for adverse events ≥ Grade 3)

Event	Anlotinib + TQB2450 (N = 34)		
	All Grades, n (%)	Grades 1–2, n (%)	Grade ≥3, n (%)
Alanine aminotransferase increased	18 (52.9)	17 (50.0)	1 (2.9)
Hand-foot syndrome	18 (52.9)	15 (44.1)	3 (8.8)
Hypertension	18 (52.9)	13 (38.2)	5 (14.7)
Aspartate aminotransferase increased	17 (50.0)	16 (47.1)	1 (2.9)
Hypertriglyceridemia	16 (47.1)	14 (41.2)	2 (5.9)
Hypercholesterolemia	15 (44.1)	14 (41.2)	1 (2.9)
Diarrhea	14 (41.2)	11 (32.4)	3 (8.8)
Prolonged QTc	13 (38.2)	7 (20.6)	6 (17.6)
White blood cell count decreased	12 (35.3)	11 (32.4)	1 (2.9)
Neutrophil count decreased	10 (29.4)	9 (26.5)	1 (2.9)
Low-density lipoprotein increased	9 (26.5)	8 (23.5)	1 (2.9)
Mucositis oral	6 (17.6)	5 (14.7)	1 (2.9)
Loss of appetite	6 (17.6)	5 (14.7)	1 (2.9)
Weight loss	5 (14.7)	4 (11.8)	1 (2.9)
Abdominal pain	5 (14.7)	4 (11.8)	1 (2.9)
Blood bilirubin increased	5 (14.7)	4 (11.8)	1 (2.9)

AEs were summarized with descriptive statistics.

Three patients experienced adverse events (AEs) leading to a dose reduction of anlotinib, including oral mucositis, hand-foot syndrome, and loss of appetite, respectively. No patients discontinued the study treatment due to AEs.

The most common immune-related AEs (irAEs) with an incidence of ≥5% of patients included hypothyroidism (n = 9 [26.5%]), increased thyroid-stimulating hormone (n = 6 [17.6%]), hyperthyroidism (n = 3 [8.8%]), increased ALT (n = 2 [5.9%]), and increased AST (n = 2 [5.9%]). No grade ≥3 irAE occurred (Table 5).

**Table 5:** Immune-related adverse events (n = 34)

Event	All grades, n (%)	Grade 1–2, n (%)	Grade 3, n (%)
Hypothyroidism	9 (26.5)	9 (26.5)	0 (0)
Thyroid-stimulating hormone increased	6 (17.6)	6 (17.6)	0 (0)
Hyperthyroidism	3 (8.8)	3 (8.8)	0 (0)
Alanine aminotransferase increased	2 (5.9)	2 (5.9)	0 (0)
Aspartate aminotransferase increased	2 (5.9)	2 (5.9)	0 (0)
Arthritis	1 (2.9)	1 (2.9)	0 (0)
Diarrhea	1 (2.9)	1 (2.9)	0 (0)
Mucositis oral	1 (2.9)	1 (2.9)	0 (0)
Immune-related pneumonitis	1 (2.9)	1 (2.9)	0 (0)
Rash	1 (2.9)	1 (2.9)	0 (0)
Skin reaction	1 (2.9)	1 (2.9)	0 (0)
Blood bilirubin increased	1 (2.9)	1 (2.9)	0 (0)

AEs were summarized with descriptive statistics.

## Discussion

In this phase Ib trial, the combination of TQB2450 and anlotinib was well-tolerated in patients with previously treated advanced TNBC, with an ORR of 26.5% and a median PFS of 5.6 (95% CI: 2.9–7.5) months. This chemotherapy-free regimen deserves further investigation.

Previous studies have shown that ICI or antiangiogenic drug monotherapy resulted in an ORR of 5.2%–22.2% in patients with previously treated advanced TNBC.^24^^,^^25^^,^^26^^,^^27^^,^^28^ However, the combination of TQB2450 and anlotinib achieved a higher ORR (26.5%), suggesting a synergistic effect of ICI and antiangiogenic drugs. This combination strategy is also supported by other studies, with an ORR of 43.3% for camrelizumab plus apatinib^20^ and 29% for pembrolizumab plus lenvatinib.^29^ Although some studies have shown a similar or higher ORR (30.0%–41%) with ICI or antiangiogenic drug combined with chemotherapy,^8^^,^^30^ we believe that TQB2450 plus anlotinib, as a chemotherapy-free regimen, warrants further investigation due to its lower toxicity compared to chemotherapy. To the best of our knowledge, TQB2450 plus anlotinib is the first regimen that uses a PD-L1 inhibitor combined with antiangiogenetic agent in TNBC. A recent *in vitro* study (2023) showed that anlotinib and an anti-PD-L1 agent inhibited the proliferation, migration, and invasion, and increased the apoptosis of TNBC cells.^31^ Furthermore, blood samples were collected in this trial to perform further bioinformatic analysis to identify patients who could benefit from the PD-L1 inhibitor plus antiangiogenic drug, but the results are not yet mature since the median OS has not been reached.

The median PFS in this study was 5.6 months, which was higher than the 3.7 months observed with camrelizumab plus apatinib^20^ and similar to the 5.1 months observed with chemotherapy (eribulin plus gemcitabine)^32^ and 6.0 months with bevacizumab plus chemotherapy^8^ in patients with previously treated advanced TNBC. Notably, the median PFS with TQB2450 plus anlotinib in the second- or further-line setting was only slightly shorter than the 7.5 months observed with first-line pembrolizumab plus chemotherapy in KEYNOTE-355^15^ and the 7.2 months observed with first-line atezolizumab plus nab-paclitaxel in IMpassion130.^33^ These findings indicate the promising survival benefit of TQB2450 plus anlotinib and suggest the potential for this combination in frontline use.

Antibody-drug conjugates (ADCs) offer a new therapeutic approach for patients with HER2-low metastatic breast cancer. Trastuzumab deruxtecan (T-DXd), a new generation of ADCs, has been shown to prolong PFS (10.1 vs. 5.4 months) and OS (23.9 vs. 17.5 months) in patients with HER2-low metastatic breast cancer compared to chemotherapy.^34^ However, it is important to note that the participants in the DESTINY-Breast04 trial was different from that in the present study, making it difficult to directly compare the efficacy of the two therapies. As the present study enrolled TNBC patients, including those with HER2-low expression, it might indicate that TQB2450 plus anlotinib could be a treatment option for patients with HER2-low metastatic breast cancer. Further studies are needed to explore this possibility.

The incidence of grade ≥3 TRAEs was 48.4% in the dose-expansion cohort, with anlotinib having more TRAEs than TQB2450. However, these TRAEs were manageable, and the discontinuation rate due to AEs was low. The AE profile was similar to previous reports on this combination,^35^ and no new safety signals were observed. The rates of grade ≥3 AST or ALT elevation with nivolumab plus cabozantinib were higher than in the present study, possibly due to the higher rates of grade ≥3 AST or ALT elevation with cabozantinib (17% and 11%, respectively)^36^ than with anlotinib (1.6% and 4.8%, respectively).^37^ In the study on camrelizumab plus apatinib, capillary hemangiomas were associated with camrelizumab.^20^ Furthermore, a meta-analysis showed that PD-L1 inhibitors were associated with a lower mean incidence of grade <3 AEs than PD-1 inhibitors.^38^ Therefore, the regimen containing TQB2450 might have fewer TRAEs, but phase III trials are necessary to confirm the safety of TQB2450 plus anlotinib.

In conclusion, TQB2450 in combination with anlotinib as a chemotherapy-free treatment shows promising efficacy and manageable safety profile in patients with previously treated advanced TNBC. Further randomized controlled trials comparing TQB2450 plus anlotinib vs. nab-paclitaxel are necessary to confirm the efficacy and safety of this regimen in advanced TNBC, especially in PD-L1-positive patients.

### Limitations of the study

There are some limitations to this study. The sample size was small, and a control group was not included for direct comparison. The numerical difference in median PFS between PD-L1-positive and PD-L1-negative patients might be due to the small sample size and needs further exploration in future studies. The sample size for the PD-L1 subgroup analysis was too small (five PD-L1-positive and 13 PD-L1-negative patients) for meaningful statistical analysis, and the Kaplan-Meier curves were crossing. Therefore, the subgroup results were purely exploratory, and no conclusion could be reached at this point. Additionally, the median OS was not reached due to the short follow-up time, but the patients are still being followed up, and data will be available in the future. As a phase Ib trial, this study had a small number of participants and short follow-up, and the results will have to be confirmed in future phases II and III trials to provide definitive efficacy and safety data for TQB2450 plus anlotinib in patients with advanced TNBC

## STAR★Methods

### Key resources table

**Table:** 

REAGENT or RESOURCE	SOURCE	IDENTIFIER
**Software and algorithms**		

SAS 9.4	SAS	https://www.sas.com/

### Resource availability

#### Lead contact

Further information and requests should be directed to and will be fulfilled by the lead contact: Binghe Xu (xubinghe@medmail.com.cn).

#### Materials availability

This study did not generate new materials.

### Experimental model and subject details

The study was a multicenter, multicohort phase Ib clinical trial. Patients were included if they were 18-75 years; histologically or cytologically confirmed recurrent or metastatic TNBC; Eastern Cooperative Oncology Group (ECOG) performance status of 0-1; expected survival of more than 3 months; at least one measurable lesion according to the Response Evaluation Criteria In Solid Tumors (RECIST) version 1.1; received at least one line of systemic therapy; prior use of anthracyclines and/or taxanes; disease progression after the last therapy; normal organ function. ER- and PR-negative was defined as an expression of ER and PR in <1% of the tumor cells by immunohistochemistry (IHC), while HER2-negative was defined as a score of 0 or 1+ by IHC, or IHC 2+ and fluorescent *in situ* hybridization (FISH)-negative. The key exclusion criteria were prior use of VEGFR-targeted inhibitor drugs or ICIs; antitumor therapy within 4 weeks prior to the initiation of treatment; uncontrollable central nervous system metastases within 8 weeks prior to the initiation of treatment; autoimmune disease.

The study was conducted in accordance with the Declarations of Helsinki and was approved by the Ethics Committee of the Cancer Hospital of the Chinese Academy of Medical Sciences (approval no. 19-001/1786). Written informed consent was obtained from all study subjects. The study was registered on ClinicalTrials.gov (NCT03855358).

### Method details

#### Procedures

In this study, two phases were conducted: the dose-escalation phase, followed by the dose-expansion phase. During the dose-escalation phase, patients were administered intravenous TQB2450 1200 mg on day 1 and oral anlotinib once daily on days 1-14 of each 21-day cycle. The dose-escalation phase consisted of three dose groups according to the dose level of anlotinib: TQB2450 1200 mg plus anlotinib 8 mg (dose level 1), TQB2450 1200 mg plus anlotinib 10 mg (dose level 2), and TQB2450 1200 mg plus anlotinib 12 mg (dose level 3). A traditional 3+3 design was used to observe the DLT and determine the maximum tolerated dose (MTD).

Based on the safety data from previous large-scale clinical studies of anlotinib, the dose-escalation phase was planned to initiate from dose level 2. In case more than 1/6 of the patients experienced a DLT, the dose level 1 group was used to explore tolerance. If dose level 1 was not well-tolerated, the study would be suspended, and the dose level would be redefined.

During the dose-expansion phase, patients were treated with the MTD determined during the dose-escalation phase until disease progression or intolerable toxicity. The drug's safety at the MTD was further evaluated, and the preliminary efficacy of this regimen was observed.

During the study, dose delay (for both study drugs) or adjustment (for anlotinib only) was allowed if necessary. The dose of anlotinib could be increased from 10 mg at the beginning of the study to 12 mg or reduced to 8 mg based on the patient's condition. For patients with a dose reduction of anlotinib to 8 mg, the dose level could be increased to 10 mg only once if the investigator determined that the disease might progress and the patient's condition was stable. Patients were required to discontinue anlotinib if they could not tolerate the 8 mg dose level or if there was a delay of anlotinib for more than 4 weeks due to TRAEs.

Delayed administration was permitted for TQB2450 instead of dose adjustment. If TQB2450 administration was delayed due to TRAEs and could not be resumed for more than 12 weeks, it was discontinued.

#### Endpoints

The primary endpoints were ORR and safety. The secondary endpoints were the PFS, DCR, DoR, 6-month PFS rate, and OS.

Efficacy was assessed according to RECIST 1.1, and patients were assessed by iRECIST when disease progression occurred. The efficacy evaluations included imaging evaluations and analyses of tumor markers.

AEs were graded per the National Cancer Institute Common Terminology Criteria for Adverse Events (CTCAE) version 5.0 throughout the study treatment and for up to 90 days after the last dose of the study drug.

### Quantification and statistical analysis

Demographics, ORR, DCR, and AEs were summarized with descriptive statistics. The Clopper-Pearson method was used to calculate the 95% confidence intervals (CIs) of ORR and DCR. PFS and OS were estimated using the Kaplan-Meier method. All patients in the dose-escalation and dose-expansion phases were included in the efficacy and safety analyses, and statistical analyses were conducted using SAS 9.4 (SAS Institute Inc., Cary, NC, USA).

#### Sample size

This study was a phase Ib dose escalation and dose expansion study. The optimal dose was determined by the "3+3 design" in the dose escalation phase. Then, 30 patients were enrolled at the optimal dose. The table below shows the two-sided 95% CI of ORR with 30 subjects for various observed response rates.

Two-sided 95% Confidence Interval of ORR with 30 Subjects

**Table:** 

Number of Observed Responders	ORR Estimates (%)	95% CI of ORR (%)
2	6.67	(0.82, 22.07)
4	13.33	(3.76, 30.72)
6	20.00	(7.71, 38.57)
8	26.67	(12.28, 45.89)
10	33.33	(17.29, 52.81)

### Additional resources

This clinical trial was registered at ClinicalTrials.gov, Identifier: NCT03855358. https://www.clinicaltrials.gov/ct2/show/NCT03855358.

## Data Availability

This article includes all data generated or analyzed during this study. This paper does not report the original code. Any additional information required to reanalyze the data reported in this paper is available from the lead contact upon request.
