# Supplementary material for: A phase Ib study of TQB2450 plus anlotinib in patients with advanced triple-negative breast cancer
Source: iScience. 2023 May 13;26(6):106876. doi: 10.1016/j.isci.2023.106876 (PMC10238930; doi:10.1016/j.isci.2023.106876)
Supplement: Document S1. Figure S1 [file mmc1.pdf]

iScience, Volume *26*

Supplemental information

A phase Ib study of TQB2450 plus anlotinib in patients with advanced triple-negative breast cancer

Jiayu Wang, Tao Sun, Quchang Ouyang, Yiqun Han, and Binghe Xu

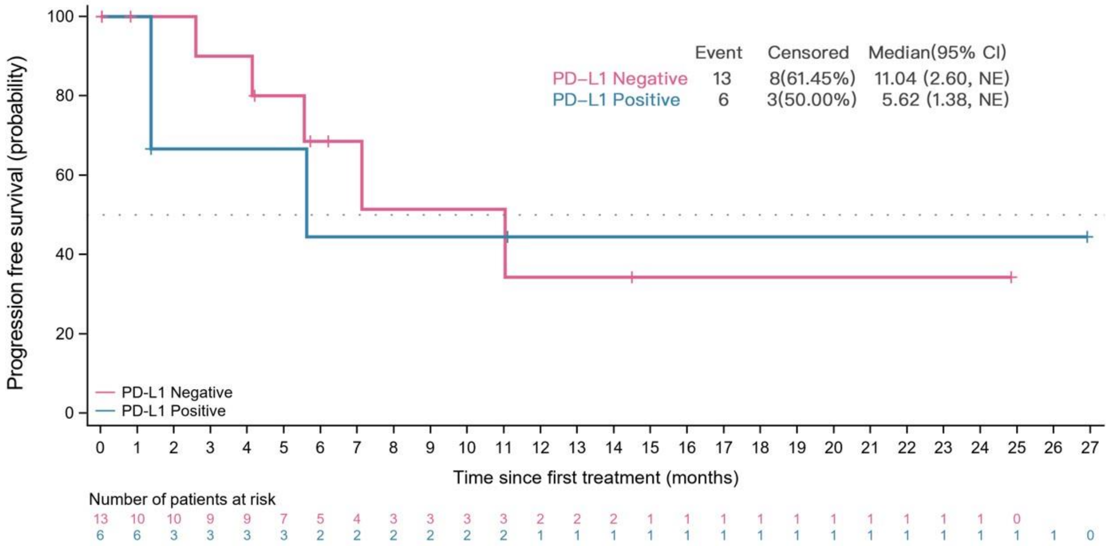

Figure S1. Kaplan-Meier curve of progression-free survival (PFS) in PD-L1 positive patients and PD-L1 negative patients, related to figure 2. PFS was estimated with the Kaplan-Meier method.
